# Generics in transplantation medicine: Randomized comparison of innovator and substitution products containing mycophenolate mofetil 

**DOI:** 10.5414/CP203487

**Published:** 2019-08-09

**Authors:** Bruno Reigner, Susan Grange, Darren Bentley, Ludger Banken, Markus Abt, Richard Hughes, Emmanuel Scheubel, Theodor W. Guentert

**Affiliations:** 1 Clinical Pharmacology, Roche Pharma Research and Early Development, Roche Innovation Center Basel,; 2 Biostatistics,; 3 Global Product Development Medical Affairs, F. Hoffmann-La Roche Ltd., Basel, and; 4 PK-Insights GmbH, Boeckten, Switzerland; a Current affiliation: Integrated Drug Development, Certara Strategic Consulting, London, UK,; b Retired,; c Current affiliation: Safety Risk Management, F. Hoffmann-La Roche Ltd., Basel, Switzerland

**Keywords:** mycophenolate mofetil, generic, pharmacokinetics, switching, equivalence

## Abstract

Objective: Mycophenolate mofetil (MMF) is widely used as an immunosuppressant for the prophylaxis of acute organ rejection in recipients of solid organ transplants. Materials and methods: We have compared, in healthy subjects, the pharmacokinetics of mycophenolic acid when MMF was administered in the form of the innovator product CellCept (F. Hoffmann-La Roche Ltd.) or one of three commercially available generics, Renodapt (Biocon Ltd.), Mycept (Panacea Biotec), or Cellmune (Cipla Ltd.). The study was powered to detect a 20% difference in mean formulation performance measures, but not to formally evaluate bioequivalence. Geometric mean ratios of maximum concentrations (C_max_) and areas under plasma concentration-time curves were calculated. Results: Comparing generics against each other, the differences in point estimates of the geometric mean ratios of C_max_ of two of the comparisons were either borderline within (Renodapt/Cellmune) or clearly outside (Mycept/Cellmune) a region of 80 – 125% around the reference mean, indicating that bioequivalence between these generics may be difficult to show. Conclusion: Physicians in the field of transplantation should be aware of the potential risk of altering the therapeutic outcome when switching from one preparation of MMF to another. ClinicalTrials.gov identifier: NCT02981290.

**What is known about this subject**

- Mycophenolate mofetil (MMF) is available as the innovator product (CellCept) and in several generic forms.
- Approval of generic MMF products has been based on pharmacokinetic bioequivalence shown between the innovator and each generic product.
- The generic products have not been directly compared with each other in bioequivalence studies.

**What this study adds**

- Comparison between generic MMF products suggests there is sufficient variation that they cannot be considered bioequivalent with each other.
- The data indicate that physicians should be cautious when considering switching between different formulations of MMF.

## Introduction 

Generic substitution is a key issue in transplantation medicine because transplant drugs are expensive and often need to be taken life-long, and because the consequences of poor immunosuppression are severe and can be life-threatening. In recent years, generic substitutes for many key immunosuppressants have entered the market and are often used interchangeably with the innovator product [1]. 

The use of generics in transplantation medicine remains a matter of debate because of the risk of graft rejection in case of treatment with a substitution product that may not be therapeutically equivalent to an innovator product [1]. Mycophenolate mofetil (MMF), the prodrug of the active mycophenolic acid (MPA), is an integral part of immunosuppressant therapy for the prophylaxis of acute organ rejection in patients receiving renal, cardiac, or hepatic transplants. Generic formulations of MMF have now received regulatory approval in various countries. According to US and EU standards, approvals are based on demonstration of bioequivalence between each individual MMF generic and the innovator product CellCept [2, 3]. However, there are no data available on the performance of generic MMF formulations against each other, even though switches from one generic to another are common. 

A study was performed in healthy subjects to compare the pharmacokinetics of MPA and its inactive 7-O-glucuronide metabolite (MPAG) following administration of CellCept or 3 commercially available generic products, selected after in vitro dissolution screening of 14 generic MMF preparations [4]. The objective was to assess in vivo possible differences between formulations, but not to formally establish bioequivalence. 

## Materials and methods 

### Study design 

This single-center, randomized, open-label, 4-treatment, 4-period, 4-sequence crossover study compared the pharmacokinetics of MMF metabolites from four tablet formulations, with at least a 7-day washout period between treatments. 

The study was conducted at Christchurch Clinical Studies Trust, Christchurch, New Zealand, in accordance with the Declaration of Helsinki, local laws and Good Clinical Practice guidelines, and all subjects gave written informed consent. The protocol (study WP21980, F. Hoffmann-La Roche Ltd., Basel, Switzerland) and accompanying material were approved by an Independent Ethics Committee. The study is registered at clinicaltrials.gov (NCT02981290). 

### Study population 

The study was performed in healthy males aged 18 – 55 years with a body mass index 18 – 32 kg/m^2^, no clinically significant hypersensitivities, and no history of substance abuse. Subjects smoking ≥ 5 cigarettes/day or equivalent or consuming > 8 caffeinated beverages/day, with positive tests for human immunodeficiency virus of hepatitis A or B virus, or with any condition likely to affect drug absorption, were excluded. No other medicines were permitted from 2 weeks before the study. 

32 subjects were enrolled to ensure at least 28 with pharmacokinetic data. In a previous study involving administration of a single 1-g dose of MMF, the intrasubject coefficients of variation (CVs) for the MPA area under the plasma concentration-time curve extrapolated to infinity (AUC_inf_) and for maximum MPA concentration occurring after an administration (C_max_) were estimated at 11% and 36%, respectively. Based on these estimates, 28 evaluable subjects provide more than 90% power for AUC_inf_ and at least 46% power for C_max_ to detect a 20% difference in means between formulations at a two-sided significance level of 0.05. 

### Medication and treatment modalities 

Three generic formulations of MMF (Renodapt, Biocon Ltd., Bangalore, India, 500 mg, batch BLMP07031; Mycept, Panacea Biotec, New Delhi, India, 500 mg, batch 0808501; Cellmune, Cipla Ltd., Mumbai, India, 500 mg, batch J75190) were sourced from commercial suppliers. CellCept tablets were obtained from F. Hoffmann-La Roche Ltd. (500 mg, batch M1916B02). 

Subjects were randomized according to a Williams Latin Square design between August 13 and September 9, 2008. Treatment sequences were ABCD, BDAC, CADB, DCBA (A Renodapt, B Mycept, C Cellmune, D CellCept). The list of randomized treatment sequence assignments was generated by F. Hoffmann-La Roche Ltd. The randomized treatment sequence assignments were allocated from the list sequentially to subjects in the order in which they enrolled. 

In each treatment period, subjects received a single 500-mg oral dose of one of the MMF formulations, with water after an overnight fast and within 10 minutes of completion of a high-fat, high-calorie breakfast. Lunch and dinner were served 4 hours and 10 hours after drug administration, respectively. Water was allowed without restriction except 1 hour before and after study drug intake. In each period, subjects were resident in the center from the day before dosing until 48 hours thereafter. 

### Safety assessments 

Safety and tolerability were assessed by adverse events, physical examination, laboratory tests/assessments, vital signs, and electrocardiogram (ECG). 

### Pharmacokinetic assessments and parameters 

Blood samples (2.0 mL) were collected into EDTA tubes over a 48-hour period after each drug intake (before dosing, then every 20 minutes for the first 2 hours, every 30 minutes for the next 3 hours, then at 6, 8, 10, 12, 16, 24, 36, and 48 hours postdose). 

All plasma samples were analyzed for MPA and MPAG at Eurofins Medinet Laboratory in The Netherlands by a specific validated liquid chromatography-mass spectrometry method. The lower limits of quantification were 0.1 µg/mL (MPA) and 1.0 µg/mL (MPAG). The precision (CV) in quality-control samples for the assays ranged from 6.4 to 9.3% for MPA and from 6.6 to 8.2% for MPAG, while the accuracy in quality control samples was 92 – 98% for both MPA and MPAG. 

MPAG concentrations were adjusted for differences in molecular weight and are reported as MPA equivalents per volume of plasma. 

MPA and MPAG pharmacokinetic parameters were derived from plasma concentrations by noncompartmental methods using WinNonlin software (Enterprise version 5.2, Pharsight Corp., Mountain View, CA, USA) and included AUC calculated using the linear trapezoidal rule between time zero (predose) and the last measurable concentration-time point (AUC_last_), or extrapolated to infinity (AUC_inf_); C_max_ and time of its occurrence (t_max_); apparent oral clearance (CL/F); apparent volume of distribution (V/F); terminal elimination rate constant (k_el_); and associated half-life (T_1/2_). AUC_inf_, CL/F, and V/F values were excluded from summary statistics and analyses if > 20% of the AUC_inf_ was extrapolated. AUC_inf_, T_1/2_, k_el_, CL/F, and V/F values were not reported if k_el_ could not be estimated accurately (i.e., r^2^ < 0.7). 

### Statistical evaluation 

The primary parameters for statistical comparisons were AUC_inf_ and C_max_ of MPA and MPAG. Although not specified in the protocol, the secondary parameter AUC_last_ was also analyzed for exploratory purposes. Other pharmacokinetic parameters such as t_max_, t_lag_ (time from administration to first observable drug concentration), T_1/2_, CL/F, and V/F were considered to be secondary parameters. The predefined statistical analysis tested – separately for each primary pharmacokinetic parameter – the global null hypothesis that there is no difference between treatments A, B, and C against the alternative that differences exist. 

A mixed-effects model with fixed effects for factors “treatment” and “period”, as well as random subject effects, was used. Statistical analyses based on this model were performed for AUC_inf_, AUC_last_, and C_max_, and data were log-transformed. Estimated exposure ratios and associated 90% confidence intervals (CIs) are reported. Although the p-values of the global comparison of MPA AUC and C_max_ of the formulations exceeded the standard significance level of 0.05 (i.e., the null hypothesis could not be rejected), back-transformed relative estimates and 90% CIs for the three generic formulations and for CellCept were calculated. These CIs were not adjusted for multiple comparisons and can be interpreted only in an exploratory sense. All analyses were carried out using SAS Version 8.2 on an HP-UX platform. 

## Results 

### Study population 

Of the 32 enrolled subjects, 1 did not attend the Renodapt treatment period, but returned for the follow-up visit. All 32 were included in the pharmacokinetic and safety analyses. 

### Safety 

The incidence of adverse events was low, with fewer than 36% of subjects with an adverse event in any treatment arm; all events were mild-moderate, and there was no apparent difference in the incidence of events between treatments. No deaths, serious adverse events, or adverse events leading to premature withdrawal were recorded. There were no clinically significant untoward laboratory findings or ECG abnormalities, and no meaningful changes in vital signs. 

### Pharmacokinetics 

**Figure 1.**
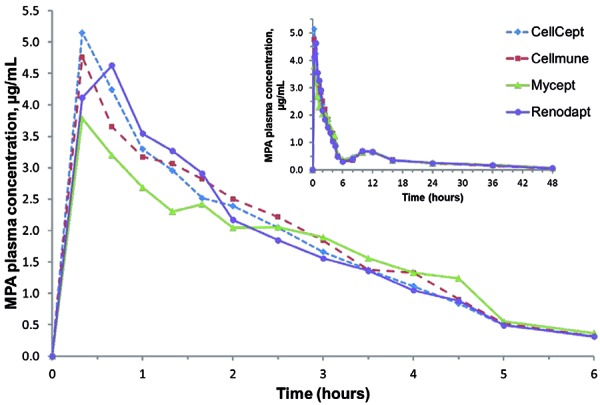
Mean MPA plasma concentration vs. time profiles (0 – 6 hours; inset: 0 – 48 hours). MPA = mycophenolic acid.

MPA plasma concentrations vs. time profiles were similar overall for all four treatments, but differences were apparent during the first 6 hours after administration (Figure 1). 

**Figure 2.**
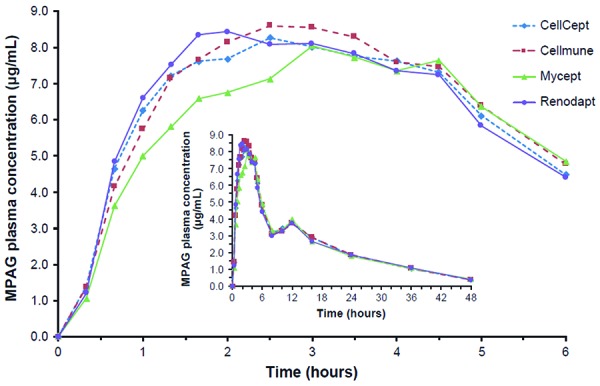
Mean MPAG plasma concentration vs. time profiles (0 – 6 hours; inset: 0 – 48 hours). MPAG = 7-O-glucuronide metabolite of mycophenolic acid.

Comparison of the MPA pharmacokinetic parameters between the three generics (Table 1) showed apparent differences in peak MPA exposure (C_max_), but fewer in overall exposure (AUC). C_max_ from Mycept tablets was on average 23% and 19% lower than from Cellmune and CellCept tablets, respectively. Peak MPA concentrations also occurred later for Mycept and Cellmune compared with CellCept. Differences in overall exposure (AUC_inf_, AUC_last_) were minor between all four preparations, and T_1/2_ of MPA after administration of all four preparations showed similar mean values and variability (Table 1), indicating that differences between products occurred mainly in absorption kinetics. Comparison of MPAG pharmacokinetics showed hardly any apparent differences between preparations (Figure 2) (Table 2). 

In the predefined statistical analysis, the p-value of the global comparison of MPA AUC and C_max_ of the formulations exceeded the standard significance level of 0.05. The null hypothesis that there were no treatment differences amongst the three generic formulations and between the generics and CellCept could not be rejected. The back-transformed relative estimates and 90% CIs for the three generic formulations and for CellCept are presented in Table 3. Residual variability in the mixed-effects model was very similar for all four formulations. Predose concentrations for MPA and MPAG for each subject across all four periods and preparations were below the limit of quantification, indicating absence of a carryover effect in the crossover design; period effects were adjusted for in the analyses by adding “period” as a factor in the model. 

In all comparisons across the three generics, the mean ratios of AUCs were close to unity, indicating that, on average, similar exposures (AUC_inf_, AUC_last_) to MPA could be achieved by these preparations, and that these were no different from the exposure after CellCept (Table 3). However, the three generics differed in their point estimate for C_max_ ratios, with large between-subject variabilities (Table 1). Such differences between generics were particularly pronounced in comparisons with Cellmune because of the lower C_max_ seen with Renodapt and Mycept. In terms of mean peak (C_max_) and average exposures (AUC measures), Cellmune performed closest to CellCept. This is confirmed by comparing their mean exposure ratios. However, in all comparisons across the three generics, the 90% CI for mean C_max_ ratios extended beyond the 80 – 125% region. The comparison of Mycept with Cellmune revealed particularly poor results, but results from Renodapt compared with Cellmune were only slightly better (Table 3). The same observation of failure to meet the bioequivalence criteria can be made for the comparisons between each of the three generic preparations with CellCept. The difference in C_max_ measures was particularly pronounced when Mycept (with its lowest mean C_max_ value) was compared with CellCept. The point estimate for the mean ratio of C_max_ values for Mycept against CellCept was barely in the equivalence region, and the 90% CI did not include unity (Table 3). The performance of Renodapt compared with CellCept was only slightly better. 

For MPAG, in all comparisons of AUC_inf_, AUC_last_, and C_max_, the geometric mean ratios between treatments were close to unity, and 90% CIs were narrow, falling into the region of 80 – 125% (Table 3). 

## Discussion 

In 2013, eleven generic MMF formulations had been approved by the US FDA [5]. Approvals are based on demonstration of bioequivalence between each product and CellCept, but there are no data required, nor any available, on the relative performance of generic formulations against each other. 

**Figure 3.**
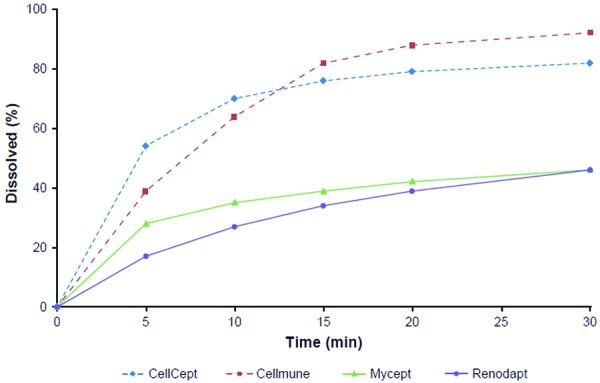
Dissolution profiles of the four MMF tablets in acetate pH 4.5 buffer at 50 rpm (adapted from Scheubel et al. [4]). MMF = mycophenolate mofetil.

The objective of the present exploratory study was to compare the pharmacokinetics of MMF metabolites from selected generic 500-mg tablet formulations against each other and against CellCept in healthy subjects. As can be seen from Figure 3, two of the preparations tested (Renodapt, Mycept) had a dissolution rate clearly inferior, and one (Cellmune) was comparable, to CellCept. 

Similar to other bioavailability assessments with MMF [6], the present study was conducted in healthy subjects. As has been noted previously [6, 7], important differences in outcome may occur between studies in healthy subjects and those in patients, and therefore results obtained in healthy subjects must be confirmed in patients. In the case of MMF, comparisons of the pharmacokinetics of MPA in stable renal transplant patients with those of healthy subjects show very similar behavior of the drug [8]. These observations suggest that our results obtained on drug formulation performance in healthy subjects are relevant for patients who are stable and have a well-functioning graft. The fact that our data are based on intraindividual comparisons further strengthens this conclusion. 

### Comparison of the three generics with each other 

The three generic preparations tested showed differences when compared with each other. While 90% CIs of mean ratios of AUC parameters were within the 80 – 125% region (i.e., the region set by regulators to indicate bioequivalence), all the 90% CIs for point estimates for C_max_ ratios of the Renodapt, Mycept, and Cellmune preparations extended beyond the region of 80 – 125%. The estimate for the C_max_ comparison between Renodapt and Cellmune (85%) was substantially lower than 100%; for the comparison of C_max_ between Mycept and Cellmune, the point estimate (77%) was not included in the 80 – 125% region. The sample size estimates (for a hypothetical bioequivalence trial between Mycept and Cellmune – up to 10,080 subjects with a classical design, up to 897 subjects with a reference-replicated design) indicate that an adequately powered bioequivalence trial would not be feasible, even with an optimized design. Pharmacokinetic studies comparing several generic substitutes for the same innovator drug are not required by regulators and are thus not performed. However, our study provides clinical data that support the validity of concerns discussed previously among policymakers and in the transplant community [9, 10, 11], namely that generics that are bioequivalent with an innovator drug may not be bioequivalent with each other. 

### Relative performance of the three generics in comparison to CellCept 

In our study, MPA AUC ratios and their 90% CIs for all generics vs. CellCept (Table 3) were within a region of 80 – 125%. Point estimates for MPA C_max_ ratios for Renodapt and Mycept were 89% and 81%, respectively, and their 90% CIs extended beyond the 80 – 125% limit. As the current study was not powered to demonstrate bioequivalence, these C_max_ results outside the accepted bioequivalence range do not necessarily indicate that the generics cannot be bioequivalent with CellCept. However, based on the differences and the intrasubject variability as derived from the residual variability in the mixed-effects model used in the present study [12], the sample sizes that would be needed to demonstrate bioequivalence with different study designs can be calculated (Table 4). The sample size estimates, up to 1,086 subjects for Mycept vs. CellCept, indicate that demonstrating bioequivalence with a classical approach would be very challenging. Results from bioequivalence trials including Renodapt or Mycept qualifying them as acceptable generic substitutes are not published in the public domain, so that our hypothesis on the likely difficulties in ascertaining bioequivalence of these products with CellCept cannot be verified. 

A potential lack of equivalence between generic substitution products has been discussed before [13, 14, 15]. Monte Carlo simulations confirmed that two generic formulations that meet regulatory approval requirements for bioequivalence with the innovator product may not be bioequivalent to one another [13, 16]. The chance for a generic G2 not to be bioequivalent with another generic G1 increases if G1 and G2 show point estimates for equivalence parameters deviating from the innovator product in the opposite direction, as shown for C_max_ between Mycept and Cellmune in our study. The simulation studies showed that switches between bioequivalent generics could lead to larger changes in plasma levels and exposures than the innovator-generic switch. Unscaled bioequivalence criteria permit the greatest dissimilarity between different formulations associated with low within-subject variability [17]. 

### Highly variable drugs: Complexities and regulatory guidance for bioequivalence assessment 

Studies with replicate designs (repeat administrations) revealed high within-subject variability in maximum concentrations (C_max_) of MPA achieved after MMF dosing; CV estimates exceeding 30% have repeatedly been shown for CellCept and for some generics [18, 19]. Corresponding variabilities in MPA AUC were lower (< 20%). In the present study, the residual variability for C_max_ in the mixed-effects model was 48.6%, confirming high variability in C_max_. A review of submitted bioequivalence studies at the US FDA Office of Generic Drugs concluded that 61% of highly variable drugs reviewed in 2003 – 2005 were likely variable due to drug substance pharmacokinetic characteristics and indicated that extensive presystemic metabolism was the most important explanation for high variability [20]. This condition applies directly to CellCept: MMF is a prodrug and activation relies on presystemic de-esterification. The within-subject variability in one of the pharmacokinetic parameters for MPA exceeding 30% classifies MMF as a high-variability drug (HVD) [21] and explains the complexities and many failures in bioequivalence testing with MPA-generating drugs [20, 22, 23, 24]. A survey from the databases of two large Canadian contract research organizations showed that the failure rate to demonstrate bioequivalence with HVDs was as high as 54% [25]. 

Using the standard average bioequivalence method to demonstrate bioequivalence in the case of HVDs requires a large number of subjects – even for test products that are bioequivalent to the reference product [20]. In published bioequivalence studies with MMF, more than 100 subjects per study had to be included to demonstrate bioequivalence [19, 26]. Sample-size calculations for a confirmatory bioequivalence trial with the generic preparations included in our study – assuming variabilities as observed here – revealed that, in the absence of a true difference between the preparations, and using the traditional average bioequivalence testing approach, at least 76 subjects (162 subjects in presence of a true difference of 10%) would be needed to show bioequivalence in both equivalence parameters between the generics used in our study. If equivalence was tested using a reference-replicated approach proposed for highly variable compounds by the EMA or US FDA guidelines (see below), only 24 – 27 subjects would need to be studied when assuming absence of a true difference between preparations (Table 4). If the generics were to be compared with each other, assuming the true differences between preparations as observed in our study, hundreds of subjects would need to be included in such testing unless one of the reference-replicated approaches were to be applied. The same would be true for adequately powered bioequivalence comparisons between each generic and CellCept [27, 28]. 

HVDs are generally assumed to have wide therapeutic windows [29]. However, this does not apply to MMF. MMF exerts its therapeutic effect as long as the MPA concentration exceeds a minimum threshold value during the interdose interval, so that an interdose AUC in the target range for MPA of 30 – 60 mg×h/L (in renal transplant recipients cotreated with cyclosporine) is maintained [30]. 

Improved designs for bioequivalence studies with HVDs have been explored over the past decade [31, 32]. Both the EMA and the US FDA have come up with a similar approach to lower the required number of subjects in bioequivalence studies with HVDs [2, 3, 32, 33], yet their solutions differ slightly [27]. The approach requires a reference-replicated design (3-period or 4-period crossover) to allow estimation of the within-subject variability for bioequivalence metrics of the reference product and to assess whether it exceeds a threshold value of 30%, indicating applicability of the procedure for HVDs. A primary advantage of this procedure is that a fixed sample size of 36 subjects is adequate to demonstrate bioequivalence, regardless of within-subject variability [25]. 

Compared with the classic 80 – 125% or the extended 75 – 133% criteria, the new EMA limits in the reference-scaled bioequivalence approach are more liberal at high intraindividual variabilities and allow greater differences between two products declared bioequivalent [34]. Hence, the reduced producer risk (that is, the risk of not being able to show bioequivalence for truly equivalent products) comes at the expense of an increased consumer risk (the risk of obtaining a generic erroneously declared bioequivalent with a reference product). The new rules set by the European bioequivalence guideline for HVDs bear the risk that a relevant number of HVDs will enter the market that would have been assessed as nonbioequivalent under the standard evaluation method [35]. 

To our knowledge, the reference-scaled bioequivalence approach for HVDs, put into effect in 2010 by the EMA and in 2011 by the US FDA, has not been applied so far to bioequivalence testing with any new MMF formulation, although this approach would ensure improved true “switchability” between two generics that are both bioequivalent with the innovator product [17]. When the average bioequivalence approach failed to show bioequivalence in C_max_, then unimportance of the deviations observed in this parameter was declared [22]. 

### Bioequivalence, switchability, therapeutic equivalence 

Traditional “average bioequivalence” testing determines whether the average values for the pharmacokinetic measures determined after administration of the test and reference products are comparable [36]. Bioequivalence relating to the mean of the data for the study population does not preclude the possibility that values for individual subjects may lie outside the bioequivalence intervals (see, e.g., cyclosporine [15]). Although this conventional bioequivalence method is considered adequate by the US FDA for most orally administered, systemically active drugs, deficiencies in today’s process to test equivalence for special categories of compounds have long been recognized [31, 37]. 

The average bioequivalence approach gives confidence in the population safety and efficacy of the test product, so that it can be prescribed to a naive patient [38]. However, it does not allow the conclusion that a patient can be transferred from one preparation to another with no change in therapeutic outcome [10]. Such “switchability” is only ensured if preparations are equivalent, not only in the population, but also in an individual [39]. For this, within-subject variability of preparations needs to be addressed, and repeated administration of preparations to an individual is required (replicate designs). 

Bioequivalence, even if established, does not necessarily equate to therapeutic equivalence, although it is implied by today’s bioequivalence regulations. A number of factors influencing the pharmacokinetics of immunosuppressive medications in solid organ transplant recipients may be different from those in healthy subjects typically included in bioequivalence testing [10, 38, 40], but are not addressed under current licensing requirements. A clinical study of the efficacy and safety of one of the generics included in our study, Mycept, casts doubt on its therapeutic equivalence with CellCept. Hematologic side effects were noted more frequently among patients on Mycept, and a possible interaction between Mycept and cyclosporine A was seen, which is not present with CellCept [41]. Myconol (Hanmi Pharmaceutical, Seoul, Korea), an MMF generic not included in our comparison, showed pharmacokinetics similar to those of CellCept, but a small proportion of patients experienced agent-specific side effects with Myconol [42]. 

Several studies have demonstrated a relationship between MPA exposure and clinical effectiveness in the prevention of acute organ allograft rejection [43, 44, 45]. Therapeutic drug monitoring has been proposed as a way of improving the outcome of MPA-based therapies [46, 47]; such monitoring may be particularly indicated when switching combination therapy or to ensure adequate immunosuppression in patients with high immunologic risk (e.g., risk of rejection, addition or removal of an interacting medication). A number of limited sampling strategies to ensure clinical feasibility of determining exposure (AUC) over a dosing interval have been proposed for various adult and pediatric patient groups [48, 49, 50]. These strategies must be tailored for the dosing regimen employed, transplant type, and the MPA-generating formulation (MMF or enteric-coated mycophenolate sodium) [47, 48]. 

### Clinical practices and concerns with generic substitution of immunosuppressants 

Economic pressure appears to mandate generic substitution, even though several investigations have shown that true savings from reduced medication acquisition costs may be offset by greater total healthcare costs caused by switching from an innovator to a generic product [17, 51, 52, 53]. Generic substitution can occur when a pharmacist refills the prescription with a different formulation [10, 11, 12]. Reasons for such substitution may include purchasing contracts, requirements from third-party payers to use the least expensive product, and unavailability of the product from a specific manufacturer. Hospital formularies often restrict medication selection to one brand that may not reflect the choices available in the community. Product switches therefore often occur when patients are admitted to, or discharged from, a healthcare facility. With such automatic substitution, the patient may experience product inconsistency, which the treating physician may not be aware of. Also, patients may lose confidence in the success of their pharmacotherapy, reducing compliance [54, 55]. 

The Abbreviated New Drug Application offers generic drug manufacturers a cost- and time-efficient solution to prove that their generic drugs are as safe and efficacious as (bioequivalent to) the innovator reference drug. However, it does not mandate generic-generic testing. Regulators assume that, together with the determination of pharmaceutical equivalence, establishing bioequivalence allows conclusion of therapeutic equivalence [2]. So far, published evidence supporting therapeutic equivalence of generic formulations in solid organ transplantation is largely lacking, but such equivalence has been challenged in other therapeutic areas (e.g., antiepileptic drugs [37]). Given the evidence in our study that generic products cannot be assumed to be bioequivalent to each other, critical drugs like immunosuppressants should not be subject to indiscriminate substitution without proof, not only of innovator product-generic, but also of generic-generic bioequivalence. Patients stable on one form of an immunosuppressant should be kept on that drug; substitution, if implemented at all, should occur only under supervision of the physician. Professional bodies and the transplant community have long requested that substitution practices be regulated to ensure consistency in substitution policy and best practices [10, 11, 12, 56, 57]. To avoid product switches going unnoticed by the physician, the Dutch Transplant Society recommends patients be informed about generic substitution and educated in how to identify the different formulations of the same drug and that they should alert the transplant physician if uncontrolled substitutions are made [12]. Each switch should be followed closely and repetitive substitutions to other generic formulations avoided [11, 12]. A Canadian working group proposes that notifying the prescriber about generic substitution of critical-dose drugs in solid organ transplant recipients should become a legal requirement. Ideally, authorization should be by the prescriber [10]. 

Our findings add to the growing evidence that switching from one generic preparation of MMF to another is associated with a meaningful risk of altering the therapeutic outcome. 

## Data-sharing statement 

Qualified researchers may request access to individual subject-level data through the clinical study data request platform: www.clinicalstudydatarequest.com. Further details on Roche’s criteria for eligible studies are available here: https://clinicalstudydatarequest.com/Study-Sponsors/Study-Sponsors-Roche.aspx. For further details on Roche’s Global Policy on the Sharing of Clinical Information and how to request access to related clinical study documents, see here: https://www.roche.com/research_and_development/who_we_are_how_we_work/clinical_trials/our_commitment_to_data_sharing.htm. 

## Acknowledgment 

The authors thank the study investigators, the study participants, and their families. Support for third-party editorial assistance for this manuscript, furnished by John Carron, PhD, of Health Interactions, was provided by F. Hoffmann-La Roche Ltd., Basel, Switzerland. 

## Funding 

This study was funded by F. Hoffmann-La Roche Ltd., Basel, Switzerland. Authors who were employees of the sponsor were involved in study design, collection, analysis, and interpretation of data; in the writing of the report; and in the decision to submit the article for publication. 

## Conflict of interest 

Bruno Reigner, Susan Grange, and Darren Bentley are employees of F. Hoffmann-La Roche Ltd. and hold stocks and stock options in F. Hoffmann-La Roche Ltd. Ludger Banken is a former employee of F. Hoffmann-La Roche Ltd. Markus Abt, Richard Hughes, and Emmanuel Scheubel are employees of F. Hoffmann-La Roche Ltd. Theodor W. Guentert is an employee of PK-Insights GmbH and a consultant for F. Hoffmann-La Roche Ltd.; he also holds stocks in F. Hoffmann-La Roche Ltd. 

**Table 1.** Summary of MPA pharmacokinetic parameters.

	Unadjusted means			
	Renodapt (n = 31)	Mycept (n = 32)	Cellmune (n = 32)	CellCept (n = 32)
AUC_last_ (µg×h/mL)	20.93 (30%)	20.42 (35%)	20.95 (28%)	21.04 (30%)
AUC_inf_ (µg×h/mL)	24.97 (27%) n = 19	28.38 (35%) n = 11	25.50 (27%) n = 16	26.33 (30%) n = 15
C_max_ (µg/mL)	5.700 (41%)	5.133 (79%)	6.647 (71%)	6.346 (58%)
t_max_ (h)	0.670 (0.33 – 3.60)	1.17 (0.33 – 4.50)	1.33 (0.33 – 4.00)	0.670 (0.33 – 4.00)
t_lag_ (h)	0.00 (0.00 – 0.33)	0.00 (0.00 – 1.67)	0.00 (0.00 – 1.00)	0.00 (0.00 – 0.00)
T_1/2_ (h)	16.18 (50%) n = 23	18.18 (57%) n = 14	14.98 (40%) n = 18	15.30 (31%) n = 17
CL/F (L/h)	20.02 (33%) n = 19	17.62 (31%) n = 11	19.60 (23%) n = 16	18.99 (23%) n = 15
V/F (L)	409.7 (37%) n = 19	385.5 (43%) n = 11	394.8 (34%) n = 16	402.4 (30%) n = 15

AUC_inf_ = area under the concentration-time curve extrapolated to infinity; AUC_last_ = area under the concentration-time curve between time zero (predose) and the last measurable time point; CL/F = apparent oral clearance; C_max_ = maximum concentration; t_lag_ = time from administration to first observable drug concentration; t_max_ = time to C_max_; V/F = apparent volume of distribution; T_1/2_ = half-life; MPA = mycophenolic acid. Geometric mean (CV%) for AUC_last_, AUC_inf_, C_max_, T_1/2_, CL/F, and V/F; median (range) for t_max_ and t_lag_. Where not all patients had sufficient data, the number of patients contributing to each parameter is stated.

**Table 2.** Summary of MPAG pharmacokinetic parameters.

	Unadjusted means			
	Renodapt (n = 31)	Mycept (n = 32)	Cellmune (n = 32)	CellCept (n = 32)
AUC_last_ (µg×h/mL)	108.0 (32%)	104.6 (39%)	108.1 (37%)	109.7 (28%)
AUC_inf_ (µg×h/mL)	124.5 (23%) n = 26	127.5 (39%) n = 22	140.1 (29%) n = 24	128.7 (26%) n = 23
C_max_ (µg/mL)	10.36 (27%)	10.28 (36%)	10.96 (35%)	10.12 (28%)
t_max_ (h)	2.00 (1.00 – 4.50)	3.00 (1.00 – 5.00)	2.50 (0.67 – 4.50)	2.50 (0.67 – 5.00)
t_lag_ (h)	0.00 (0.00 – 1.33)	0.00 (0.00 – 2.00)	0.00 (0.00 – 2.00)	0.00 (0.00 – 1.67)
T_1/2_ (h)	14.29 (29%) n = 30	14.19 (39%) n = 28	14.54 (27%) n = 31	15.55 (32%) n = 31

AUC_inf_ = area under the concentration-time curve extrapolated to infinity; AUC_last_ = area under the concentration-time curve between time zero (predose) and the last measurable time point; CL/F = apparent oral clearance; C_max_ = maximum concentration; t_lag_ = time from administration to first observable drug concentration; t_max_ = time to C_max_; V/F = apparent volume of distribution; T_1/2_ = half-life; MPAG = 7-O-glucuronide metabolite of mycophenolic acid. Geometric mean (CV%) for AUC_last_, AUC_inf_, C_max_, and T_1/2_; median (range) for t_max_ and t_lag_. Where not all patients had sufficient data, the number contributing to each parameter is stated.

**Table 3.** Estimated exposure ratio for AUCinf, AUClast, and Cmax of MPA and MPAG and 90% confidence intervals.

Analyte	Parameter	Comparison	Relative results		Analyte	Parameter	Comparison	Relative results	
			Estimate (%)	90% CI (%)				Estimate (%)	90% CI (%)
MPA	AUC_inf_	A/B	103	96 – 112	MPA	AUC_inf_	A/D	103	96 – 111
		A/C	100	93 – 107			B/D	100	92 – 108
		B/C	97	89 – 105			C/D	103	95 – 112
	AUC_last_	A/B	102	97 – 108		AUC_last_	A/D	99	94 – 105
		A/C	100	94 – 105			B/D	97	92 – 102
		B/C	97	92 – 103			C/D	100	94 – 105
	C_max_	A/B	110	91 – 134		C_max_	A/D	89	73 – 108
		A/C	85	70 – 103			B/D	81	67 – 98
		B/C	77	64 – 94			C/D	105	87 – 127
MPAG	AUC_inf_	A/B	101	96 – 106	MPAG	AUC_inf_	A/D	97	92 – 101
		A/C	98	93 – 103			B/D	96	91 – 101
		B/C	98	93 – 103			C/D	98	94 – 103
	AUC_last_	A/B	103	98 – 108		AUC_last_	A/D	98	93 – 103
		A/C	99	94 – 105			B/D	95	91 – 100
		B/C	97	92 – 102			C/D	99	94 – 104
	C_max_	A/B	100	92 – 108		C_max_	A/D	101	94 – 109
		A/C	93	86 – 101			B/D	102	94 – 110
		B/C	94	87 – 101			C/D	108	100 – 117

AUC_inf_ = area under the concentration–time curve extrapolated to infinity; AUC_last_ = area under the concentration-time curve between time zero (predose) and the last measurable time point; C_max_ = maximum concentration; MPA = mycophenolic acid; MPAG = 7-O-glucuronide metabolite of mycophenolic acid. Key: A = Renodapt; B = Mycept; C = Cellmune; D = CellCept.

**Table 4.** Sample size estimations to demonstrate average bioequivalence in studies comparing two formulations in designs without or with reference-replicated administrations, and when applying different acceptance criteria.

Type of comparison	TD	80 – 125%, no replicate (2 admin/subject)	Reference-scaled approach acc. to FDA preferred with HVD (3 admin/subject)	Reference-scaled approach acc. to EMA preferred with HVD (3 admin/subject)
	0%	76	24	27
Comparison with CellCept				
A vs. D	11%	188	30	39
B vs. D	19%	1,086	96	99
C vs. D	5%	92	24	30
Comparisons between generics				
A vs. B	10%	162	30	36
A vs. C	15%	380	42	54
B vs. C	23%	10,080	879	879

EMA = European Medicines Agency; FDA = US Food and Drug Administration; HVD = high-variability drug. Calculations of sample size done by CRAN package “Power TOST” [28], assuming that the difference in the geometric means of maximum concentration (C_max_) between preparations as observed in the present study is the true difference (TD). Calculations use 80% power and the C_max_ residual variability observed in the present study (coefficient of variation of 48.6%). Key: A = Renodapt; B = Mycept; C = Cellmune; D = CellCept.
